# Secondary and Topological Structural Merge Prediction of Alpha-Helical Transmembrane Proteins Using a Hybrid Model Based on Hidden Markov and Long Short-Term Memory Neural Networks

**DOI:** 10.3390/ijms24065720

**Published:** 2023-03-16

**Authors:** Ting Gao, Yutong Zhao, Li Zhang, Han Wang

**Affiliations:** 1 School of Information Science and Technology, Institute of Computational Biology, Northeast Normal University, Changchun 130117, China; gaot080@nenu.edu.cn (T.G.); zhaoyt699@nenu.edu.cn (Y.Z.); 2 School of Computer Science and Engineering, Changchun University of Technology, Changchun 130012, China; lizhang@ccut.edu.cn

**Keywords:** transmembrane protein, topology structure, secondary structure, merge prediction, deep learning neural networks (DNNs), class hidden markov model (CHMM), long short-term memory (LSTM) networks

## Abstract

Alpha-helical transmembrane proteins (αTMPs) play essential roles in drug targeting and disease treatments. Due to the challenges of using experimental methods to determine their structure, αTMPs have far fewer known structures than soluble proteins. The topology of transmembrane proteins (TMPs) can determine the spatial conformation relative to the membrane, while the secondary structure helps to identify their functional domain. They are highly correlated on αTMPs sequences, and achieving a merge prediction is instructive for further understanding the structure and function of αTMPs. In this study, we implemented a hybrid model combining Deep Learning Neural Networks (DNNs) with a Class Hidden Markov Model (CHMM), namely HDNNtopss. DNNs extract rich contextual features through stacked attention-enhanced Bidirectional Long Short-Term Memory (BiLSTM) networks and Convolutional Neural Networks (CNNs), and CHMM captures state-associative temporal features. The hybrid model not only reasonably considers the probability of the state path but also has a fitting and feature-extraction capability for deep learning, which enables flexible prediction and makes the resulting sequence more biologically meaningful. It outperforms current advanced merge-prediction methods with a Q4 of 0.779 and an MCC of 0.673 on the independent test dataset, which have practical, solid significance. In comparison to advanced prediction methods for topological and secondary structures, it achieves the highest topology prediction with a Q2 of 0.884, which has a strong comprehensive performance. At the same time, we implemented a joint training method, Co-HDNNtopss, and achieved a good performance to provide an important reference for similar hybrid-model training.

## 1. Introduction

Alpha-helical transmembrane proteins (αTMPs) play an important role in physiological processes and biochemical pathways, particularly in drug targeting and disease therapy [1]. The 3D structure of αTMPs are challenging to determine using nuclear magnetic resonance (NMR) and X-ray crystal diffraction [2,3] due to their structural specificity. Due to the physiological significance of αTMPs and the lack of known structures [4,5], many bioinformatics approaches have explored their structure and function based on amino acid sequences.

The protein’s secondary structure helps to identify functional domains [6,7,8]. Topological structure is generally the position of transmembrane proteins (TMPs) relative to the biological membrane and the number of transmembrane segments. The secondary structure of the transmembrane region of αTMPs is the α-helix. The combined prediction of the secondary and topological structures avoids conflicting predictions of alpha-helix transmembrane structures caused by different structural predictors. Moreover, it provides a simpler and more effective method for the local primary screening of drug-targeted binding and helps subsequent studies focus on the appropriate region of action in advance. Although Alphafold2 has been shown to perform well in predicting transmembrane proteins [9], the study of the combined structure can provide an important reference for related applications.

Machine Learning (ML) improved the performance of structural predictors, replacing earlier empirical, statistical, and principle approaches [10,11,12]. Neural Networks (NNs) were successfully applied to the secondary structure-prediction methods JPred, PSIPRED, and PredictProtein [13,14,15]. PHDhtm [7] and MEMSAT3 [16] used NNs to improve the topology prediction performance. TMHMM [17] and HMMTOP [18] applied Hidden Markov Models (HMMs) to solve topology-prediction problems. However, HMMs lack the ability to extract long-term correlations for complex patterns. OCTOPUS [19] and SPOCTOPUS [20] further improved topology-prediction performance by combining HMMs and NNs. Deep Learning (DL) offers the possibility of learning more implicit features from biological sequences. MUFOLD-SS and DeepTMHMM use DL to predict the protein’s secondary structure and topology of TMPs, respectively [21,22]. However, there are only a few methods for the merge prediction of the topological and secondary structures of TMPs. TMPSS [23] and MASSP [24] achieved simultaneous prediction by using the DL of Convolutional Neural Networks (CNNs) or Long Short-Term Memory (LSTM) layers, and such studies usually employed multi-task learning to predict structures separately, creating conflicting prediction results. Furthermore, DL is not well suited for modeling temporal phenomena.

Hidden Neural Networks (HNNs) provide new insight by reasonably combining the Class Hidden Markov Model (CHMM) and NNs [25,26]. Similar hybrid models have been applied in many fields such as speech recognition, time-perception recommendation systems [27,28,29], and such forth due to their excellent performance. HNNs have also been explored in computational biology [30,31,32]. The HNNs’ prediction performance can be further improved by choosing more complex Deep Learning Neural Networks (DNNs) instead of a simple multilayer perceptron. In this study, we implemented topology and secondary structure merge-prediction methods for αTMPs, which construct hybrid models by combining CHMM and LSTM. The hybrid models not only have the ability of general DNNs to learn complex patterns, but also constrain the corresponding state paths using statistical transition probabilities, making the results more biologically meaningful in practice. A deep learning module consisting mainly of LSTM was used to capture context dependencies of the sequence. The CHMM module extracts temporal features and correlations of states, where the final output of the deep learning module assigned to each state was used as the emission probability by the CHMM. The prediction of the topological structure corresponding to the Q2 metric refers to the accuracy of Transmembrane (T) and No-transmembrane (N) in classification. The prediction of the protein’s secondary structure corresponds to the Q3 metric refers to helix, strand, and coil. Merge prediction corresponds to Q4 and refers to T-helix (M), N-helix (H), N-strand (E), and N-coil (C). HDNNtopss, as a merged predictor which uses a separate training method, used independently generated datasets to achieve a Q4 of 0.779 in merge prediction, and it achieved a Q2 of a whopping 0.884 in topology prediction. It has strong practical significance and all-around performance as the final predictor of this study. At the same time, we put forward a joint training idea, Co-HDNNtopss, which combined DNNs and CHMM and attempted to mutually promote both to achieve the best convergence effect in training. Although the current result is not the best, potentially due to the unbalanced ratio of structural label categories, it can provide some reference for similar hybrid-model training. Our method, pre-trained models, and supporting materials can be accessed through https://github.com/NENUBioCompute/HDNNtopss (accessed on 10 March 2023).

## 2. Results and Discussion

### 2.1. Evaluations Criteria

Combined with the evaluation criteria for predicting the secondary structure and topology of transmembrane proteins at the residual level, for the combined prediction of these two structures of transmembrane proteins, we evaluated the performance of the methods based on the following metrics, where *TN*, *TP*, *FN*, and *FP*, respectively, denoted true negative, true positive, false negative, and false positive samples.

*ACC* is the number of correctly predicted residues in standard state mode, corresponding to Q2, Q3, and Q4.

(1) $$\mathrm{ACC}=\frac{TN+TP}{TP+FN+FP+TN}\;$$

To evaluate the performance of the model, we also took the following metrics, including the Matthews Correlation Coefficient (*MCC*), Segment Overlap Measure (*SOV*) [33], Recall, Precision, Specificity, and F1-score [34,35,36]. The formulae are as follows:

(2) $$MCC=\frac{TP*TN-FP*FN}{\sqrt{\left(\;TP+FP\right)\left(\;TP+FN\right)\left(\;TN+FP\right)\left(\;TN+FN\right)}}\;$$

(3) $$\mathrm{SOV}\;=\frac{100}{N_{\mathrm{SOV}\;}}\sum_{S_{0}}\left[\frac{min\left(\;S_{1},S_{2}\right)+\delta \left(\;S_{1},S_{2}\right)}{max\left(\;S_{1},S_{2}\right)}\;\mathrm{length}\left(\;S_{1}\right)\right]$$

(4) $$\delta \left(S_{1},S_{2}\right)=min\left\{\begin{array}{c} \left(max\left(S_{1},S_{2}\right)-min\left(S_{1},S_{2}\right)\right)\\ min\left(S_{1}S_{2}\right)\\ int\left[\;\mathrm{length}\;\left(S_{1}\right)÷2\right]\\ int\left[\;\mathrm{length}\;\left(S2\right)÷2\right] \end{array}\right\}\;$$

(5) $$\mathrm{Recall}\;=\frac{TP}{TP+FN}\;$$

(6) $$\mathrm{Precision}\;=\frac{TP}{TP+FP}\;$$

(7) $$\mathrm{Specificity}\;=\frac{TN}{FP+TN}\;$$

(8) $$F1-\mathrm{score}\;=2\times \frac{\;\mathrm{Recall}\;\times \;\mathrm{Precision}\;}{\;\mathrm{Recall}+\;\mathrm{Precision}\;}\;$$

The correct *TM*, indicates the number of proteins with the correctly predicted number of transmembrane strands.

(9) $$TM=\sum_{i=1}^{N}\;x_{i};\;x_{i}=\left\{\begin{array}{c} 1,\;\mathrm{if}\;PRE_{Hi}=REC_{it}=1\\ 0,\;\mathrm{else}\; \end{array}\right.$$

### 2.2. Window Size Exploration Experiment of HDNNtopss

The selection of the optimal network input size is the key to deep learning networks. Based on the above accuracy measurements, the optimal network input size of HDNNtopss was determined, and the results are shown in Table 1. Considering the accuracy of transmembrane fragment identification, the Q4, and the MCC of merge-structure prediction, a network input with a window size of 19 yields the best network-prediction results for αTMPs on HDNNtopss.

### 2.3. Prediction Performance Analysis at the Residue Level

**Figure 1:**
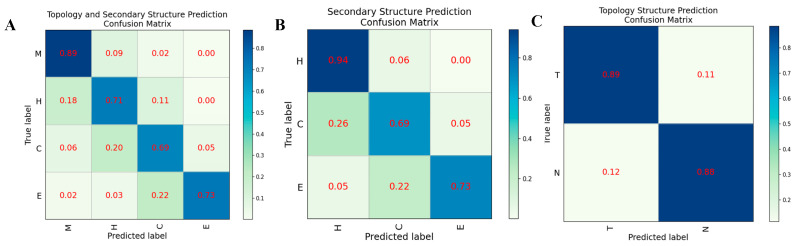
Confusion matrix on different structural predictions of HDNNtopss (**A**) Structure merge prediction. (**B**) Secondary structure prediction. (**C**) Topology structure prediction.

**Figure 2:**
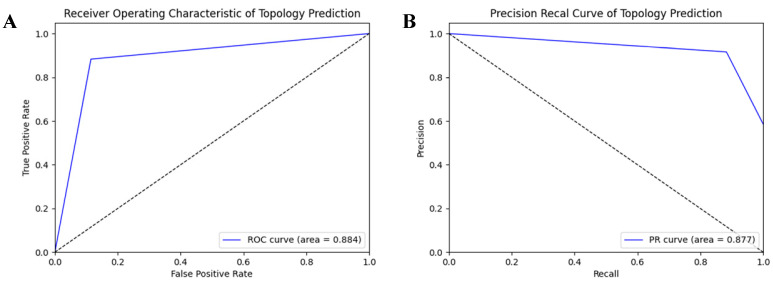
ROC and PR on topology prediction. (**A**) ROC on topology prediction. (**B**) PR on topology prediction.

In order to evaluate the prediction performance at the residual level, we used a confusion matrix, Receiver Operating Characteristic (ROC) curve, and Precision-Recall (PR) curve to display the prediction results of HDNNtopss for the blind-test set (see Figure 1 and Figure 2). Figure 1A shows the confusion matrix of the merge-structure prediction, while Figure 1B,C show the confusion matrix of the secondary-structure prediction and topology prediction, respectively. It can be seen that the topology prediction of αTMPs is good, while there are some confusing structures in the secondary-structure prediction. Figure 2A,B show the ROC and PR curves on topology prediction, respectively. They also support the conclusion.

**Figure 3:**
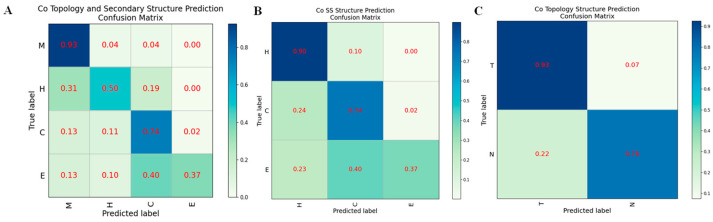
Confusion matrix on different structural predictions of Co-HDNNtopss. (**A**) Structure merge prediction. (**B**) Secondary structure prediction. (**C**) Topology structure prediction.

For Co-HDNNtopss, we showed and analyzed the performance of each structure category using the confusion matrix. It can be seen from Figure 3 that the transmembrane (T) and non-transmembrane regions (N) under the topological-structure prediction classification criteria of the C-plot can be well-divided and handled. There is not much confusion between the two categories, and only a few residues originally in the N region are predicted to be in the T region. In contrast, the B-plot, corresponding to the classification criteria for secondary-structure prediction, shows that class ‘E’ (strand) is poorly predicted, and this class is confused with both ‘H’ (helix) and ‘C’ (coil), i.e., residues originally labeled as ‘E’ are predicted as ‘H’ or ‘C’, while the box value of ‘E’ in the confusion matrix is only 0.37. This result suggests that the unevenness of the structural classes of αTMPs leads to biased prediction results. This phenomenon was not evident in the predictions of the separate training approach, suggesting that this joint training approach amplifies the defect of sample category imbalance. The A-plot, corresponding to the classification criteria of the structure merge prediction, also shows that the N-helix (H) and N-strand (E) have slightly poorer prediction results, except for the T-helix (M) and N-coil (C), which have a clearer sample category delineation.

After analysis, this was hypothesized to be due to the joint training process in which we used the sigmoid output for each category in the deep learning module for its probability, while some categories with a smaller proportion could not use the softmax output as in the separate training process, forcing each category to adjust and promote each other in time. Furthermore, the phenomenon of fewer positive samples and more negative samples creates bias in the classification model.

### 2.4. Overall Prediction Performance

To compare the accuracy in predicting the four classes of transmembrane-protein topology and secondary structures combined, we compared the results with TMPSS, which predicts both structures simultaneously. Since these two predictions are simultaneous rather than combined, we transformed the prediction of topological dichotomization and secondary-structure dichotomization to obtain four classification structures. The BioChemical Library (BCL), a necessary tool of MASSP, could not be downloaded when we tested it, and the web server provided by MASSP was not available [24]. Therefore, we didn’t compare it with another method for the multi-task prediction of two structures using MASSP.

In Table 2, the results show that the performance of our separate training predictor is better; the HDNNtopss method achieved a Q4 of 0.779 and an MCC of 0.673, while the other classification criteria also performed well. The time indicator showed the prediction time excluding the generation of HHblits profiles in the test set, and HDNNtopss consumes the least time. Whether it is due to the structural relevance of the combined prediction of the two structures or the sensitivity created by the hybrid model, our work using HDNNtopss showed the best performance. After discussion, HDNNtopss has some practical significance, and the combined prediction of structures may also provide a reference role for local primary screening for drug-target binding. Table 2 also shows the metrics of the joint training model for each category, and it can be seen that M and C are well predicted, while E is slightly worse with lower recall. The results indicate that the non-transmembrane strand with smaller proportions is poorly predicted due to the unbalanced sample categories, and the joint training method is less suitable as the final prediction model compared to the separate training method. However, it can provide a reference for similar mixed-model training, especially under data with a balanced proportion of categories.

### 2.5. Topological Structure Prediction Performance

To compare the expressiveness of topology prediction with current advanced methods, we transformed the output’s quadruple classification results into two classifications for transmembrane and non-transmembrane regions. Table 3 reports the comparative analysis of different methods in terms of topology-prediction performance. In addition to the separate training method, HDNNtopss, and joint training, Co-HDNNtopss, the state-of-the-art tested methods include TOPCONS [37], CCTOP [38], PolyPhobius [39], OCTOPUS [19], SPOCTOPUS [20], SCAMPI_MSA [40], DeepTMHMM [22], Phobius [41], Philius [42], and TMPSS [23]. For topology prediction, the methods were compared on the Test50 defined in this paper.

As seen from Table 3, by testing using an independent test set, our HDNNtopss Q2 was the best, reaching 0.884 with an MCC also reaching 0.763. It can be seen that the Q2 of our method was the same as that of CCTOP, but the MCC was slightly better. Although TM was slightly lower than DeepTMHMM, it is worth mentioning that up to six transmembrane proteins were predicted as non-transmembrane proteins using this method. Overall, our method had the best prediction ability for the topology.

### 2.6. Secondary Structure Prediction Performance

In order to compare the expressiveness of secondary structure prediction with current advanced methods, we transformed the output’s quadruple classification results into three classifications for helix, coil, and strand. Table 4 reports the comparative analysis of different methods in terms of secondary-structure prediction performance. In addition to the separate training method, HDNNtopss, and common training, Co-HDNNtopss, the state-of-the-art tested methods included JPred4 [13], SSpro5 [43], Spider3 [44], PSIPRED4.0 [45], MUFOLD-SS [21], DeepCNF [46], Porter 5 [47], and TMPSS [23]. The methods were compared on the Test50 defined in this paper.

In terms of secondary-structure prediction results, the prediction performance of SSpro5 is far ahead of other methods. Furthermore, it can be seen that the prediction of our separate training method, HDNNtopss, for the secondary structure is slightly worse compared with some current advanced methods, but it also achieves a better prediction level.

In summary, our method HDNNtopss also achieves better performance under the topology and secondary-structure prediction classification criteria and has a strong synthesis capability.

### 2.7. Case Studies

To further discuss the validity of HDNNtopss in predicting the topology and secondary structure of αTMPs, we performed a case study of several alpha-helical transmembrane proteins. By comparing the predicted labels with the real labels on the 3D view, it was found that, although most of the structures were accurately predicted by the predictor, there were still some issues that needed to be improved. The details are discussed below, where the results are visualized using PyMOL [48].

**Figure 4:**
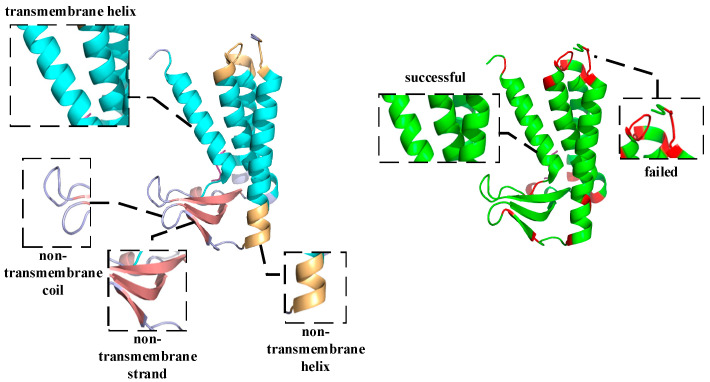
Schematic diagram of the structure of the alpha-helical transmembrane protein, 6ryo_A, and a schematic diagram of the difference between the predicted and real structures.

As shown in Figure 4, the predicted topology of 6ryo_A is shown on the left side. 6ryo is a lipoprotein signal peptidase from Staphylococcus aureus [49], and it is important for the study of antibiotic resistance. The cyan part indicates the predicted result of M, the light orange part indicates the predicted result of H, the light blue part indicates the predicted result of C, and the salmon part indicates the predicted result of E, On the right side, the difference between the predicted structure and the real structure is shown, the green part indicates a successful prediction, and the red part indicates a failed prediction. Comparing the results with the real structural labels, it can be found that the prediction model basically restores the structure of 6ryo_A, except for one to several residues at the junction that were incorrectly predicted.

**Figure 5:**
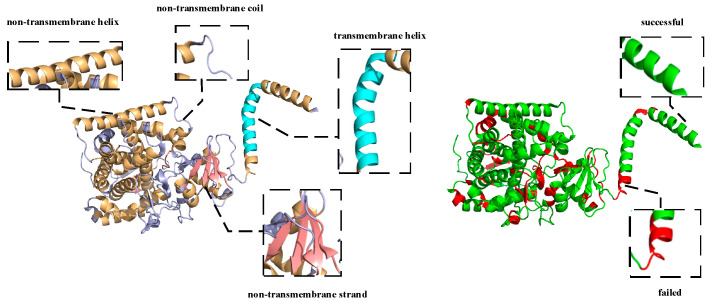
Schematic diagram of the structure of the alpha-helical transmembrane protein, 4wmz_A, and a schematic diagram of the difference between the predicted and real structures.

The vast majority of αTMPs are similar to 6ryo_A, and their topology and secondary structure can be largely recovered using this predictor with only a few errors of the poor identification of the junction of different structures. This may be caused by the different labeling criteria of the training set and the dependence of the deep learning method on the data. The analysis of a large number of results revealed that a very small number of αTMPs showed a small confusion error between different structures, except for boundary prediction error, which occurred in 4wmz_A. 4wmz exists in the Saccharomyces cerevisiae YJM789 organism, and it is a kind of oxidoreductase inhibitor [50], which provides an in-depth understanding of a drug-resistance mechanism. As shown in Figure 5 for 4wmz_A, the color-labeling and significance are consistent with Figure 4. It can be seen that, although the transmembrane fragments were correctly identified, a small segment of residue that should have been H was predicted to be C, and some that should have been C was predicted to be E. This indicates that, due to the unbalanced proportion of samples, some of the samples could not be accurately identified due to the small proportion, and a small number of unreasonable structures were not adjusted by the model. However, it can also be seen that, although the protein has only one transmembrane segment, we accurately predicted it. Although our predictor still has some small problems, it does have quite a good prediction ability.

## 3. Materials and Methods

### 3.1. Datasets

TMPs are usually large in size. Moreover, some parts are buried in biological membranes, and most of them are difficult to dissolve in appropriate detergents, inhibiting their purification and crystallization. Therefore, fewer TMPs with known structures were included in the study compared to proteins in general. The number of known structures of transmembrane proteins is increasing at a slow rate, but our predictor can provide a reference for local primary screening. We chose to process the data source to obtain the dataset. The Protein Data Bank of transmembrane proteins (PDBTM) database [51] was the data source. Approximately 5721 αTMPs were downloaded from the 13 August 2021 version of the PDBTM database. Chains containing unknown residues such as (“X”) and those <30 in length were removed. CD-HIT [52,53] was used to remove redundancy, retaining 30% sequence similarity, leaving 1242 chains. The data were randomly divided into 1142 protein chains in the training set, 50 in the test set named Test50, and 50 in the valid set. Regarding the division of the 4 classes of combined structures, for the transmembrane helix structure, we took the more accurate structure obtained using the resolution in PDBTM [51] as the standard. For the 3 classes of secondary structures in the non-transmembrane region, we took the Protein Data Bank (PDB) file analyzed using the DSSP [54] program as the standard.

### 3.2. Inputs Encoding

Based on the special structure and function of αTMPs, we chose two input-encoding features of amino acids for the deep learning module: the One-hot code and the HHblits [55] evolutionary profile. In our experiments, the HHblits used the database uniprot20_2016_02. The profile values were scaled by the sigmoid function into the range (0, 1), making the distribution more uniform and reasonable (see Equation (10)). Each amino acid in the protein sequence was represented as a vector of 30 HHblits profile values and 1 NoSeq label (representing a gap). The One-hot encoding matrix, which indicates the index of the amino acid species to which the residue belongs, was based on a 20-dimensional One-hot and a 1-dimensional NoSeq. There was a total of 52-dimensional feature encoding matrices.

(10) $$f\left(t\right)=\frac{10}{1+e^{-\frac{t}{2000}}}$$

The inputs of CHMM in the real sense are protein sequences that were encoded using numbers from 0–19 corresponding to each amino acid. The inputs conform to the observation sequence in the general HMMs. This was based on the trained transition probabilities and the emission probabilities obtained from the deep learning module used to decode sequences.

### 3.3. Models Framework

We implemented a separate training method, HDNNtopss, and a joint training, Co-HDNNtopss. They both use the hybrid model of a deep learning module and CHMM.

**Figure 6:**
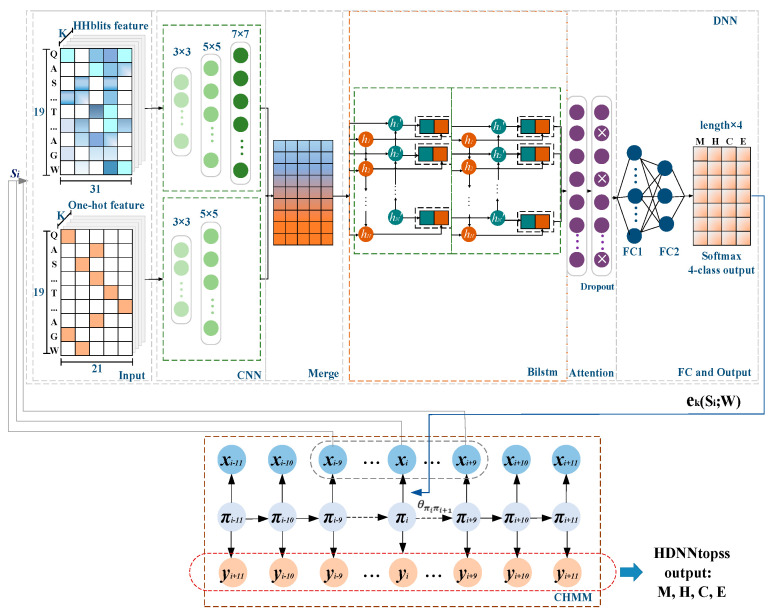
Diagram of HDNNtopss. Each amino acid in the observed sequence of αTMPs in the CHMM module of the figure is used as input in the deep learning module by encoding the contextual sliding window. The output of the deep learning module is replaced with the emission probabilities in the CHMM. Finally, the model decodes the output based on the trained parameters.

For HDNNtopss (see Figure 6), the DNN part comprises the deep learning module. Each observation in the protein sequence is encoded by contextual amino acid as input to the DNNs. The output of the deep learning module is the probability matrix corresponding to each state of the sequence observations. The emission probability of CHMM is replaced by the output to realize the combination of the two modules. CHMM uses the Viterbi algorithm [56] for optimizing the segmentation of state predictions generated by the deep learning module. The 4-category predicted merge structures are the final output.

**Figure 7:**
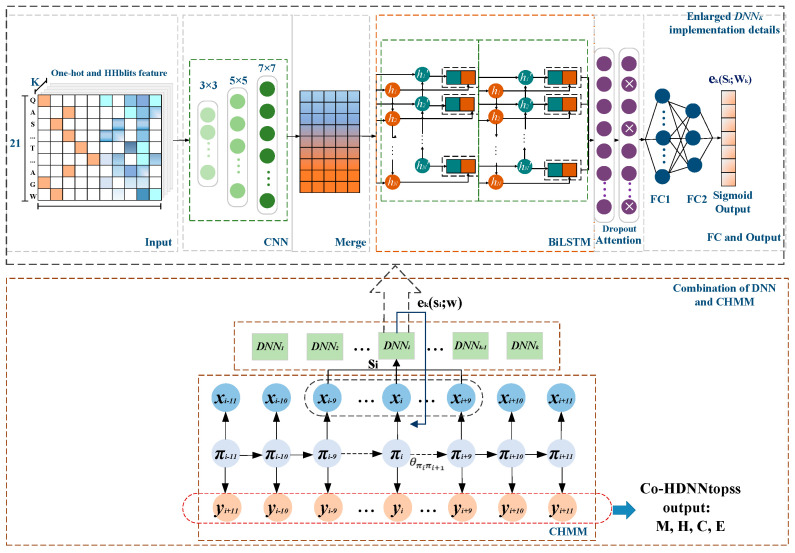
Diagram of Co-HDNNtopss. As shown in the combination of DNN and CHMM, the context $s_{i}$ of CHMM is the input for *DNN_k_*, and the initial value of each training round of the emission probability of a state in CHMM is replaced by *DNN_k_* output. The enlarged implementation details of *DNN_k_* are shown in the figure. Finally, the model decodes outputs based on the trained parameters.

For Co-HDNNtopss (see Figure 7), the combination of DNNs and CHMM shows the following details. The context $s_{i}$ of the observations from the CHMM is input to the *DNN_k_*, which outputs the values corresponding to the emission network for the state *k*. The first half shows the detailed implementation details of the *DNN_k_* emission network. In order to reduce the complexity of the model in the joint training to reduce the time consumption of training, after many experiments and attempts, the deep learning module was finally chosen; see Enlarged *DNN_k_* implementation details, in Figure 7.

### 3.4. Deep Learning Module

The deep learning module in HDNNtopss is mainly composed of CNNs and attention-enhanced Bidirectional Long Short-Term Memory (BiLSTM) layers. As shown in the DNN part of Figure 6, this includes feature-integration layers, CNNs, attention-enhanced BiLSTM layers, and fully connected layers. The input to the model is the HHBlits profile and One-hot code generated from the sequence, using a sliding window of the length of 19 residues as its central residue feature. Then, the input of the model is passed to Stacked CNN layers to extract effective data features separately, and BiLSTM layers capture the longer distance as dependent relations and global information. We also added an attention mechanism to the BiLSTM layers to help the model enhance attention to the critical regions. After the BiLSTM, there are two fully connected layers. Apart from the original HNN text, in order to avoid training the emission network of multiple groups of states, we did not use sigmoid but the softmax function as the activation function of the last layer.

The deep learning module in Co-HDNNtopss is almost the same as in the HDNNtopss method but simpler. The input part is a mixture of two features into the stacked CNNs, attention-enhanced BiLSTM with fewer units, and fully connected layers. To facilitate the rewriting of the loss function in the joint training, the sigmoid output probability of each state is treated as the emission probability in CHMM.

### 3.5. CHMM Module

CHMM is more suitable for complex protein-structure prediction problems than the standard HMM with specified labeled data [26]. The CHMM module is the same in separate training and joint training. For predicting the structure of αTMPs, assume that the protein sequence is x, a state sequence π, and a set of labels y.

**Figure 8:**
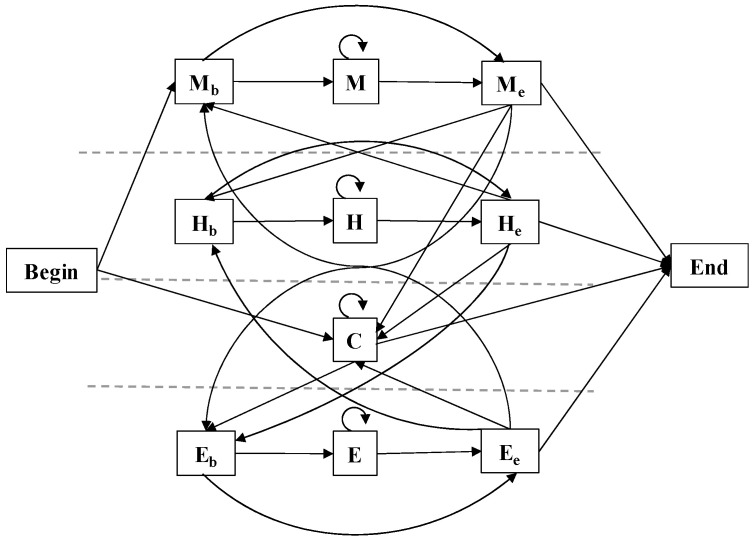
State model diagram. There are 12 states including Beginning and End states, divided into 4 sub-models, and the figure shows the possible transitions between states.

Our model consisted of 12 states, including Begin and End states, and conceptually combines the previous ideas of αTMPs topology and protein secondary-structure prediction [31,57,58]. The model consists of 4 sub-models corresponding to the 4 labels to be predicted, namely, sub-models M, H, C, and E. Except for C, each sub-model contains a beginning and end state, and the state transition of the model is shown in Figure 8.

The transition probability of CHMM from $\pi_{i}$ to $\pi_{i+1}$ is denoted by $\;\theta_{\pi_{i}\pi_{i+1}}$. The emission probability of the state $\pi_{i},$ generating the observation $x_{i},$ is denoted by $e_{\pi i}\left(x_{i}\right)$. The output value of the corresponding state of the DNNs is used to replace the emission probabilities between each observation and state. We obtained the appropriate transition probability using the supervised training of CHMM. A protein sequence x was decoded into the corresponding structural properties by using the above probability parameter.

### 3.6. HDNNtopss Module Training

HDNNtopss is a separate training method; the deep learning module uses the supervise learning method, and CHMM uses Conditional Maximum Likelihood (CML) estimation.

For deep learning modules, we used labels to supervise learning and training to get the emission value $e_{k}\left(s_{i};w\right)$ between the observation and state, where $s_{i}$ refers to the contextual encoding of each $x_{i}$, and w refers to the DNNs weights. An early termination strategy and a save-best strategy were adopted. When the loss value of the verification set did not continue to decline within the training period of 10 epochs, the best weight information of the model was retained and training was stopped. The parameters were trained using an Adam optimizer [59] to change the learning rate during model training dynamically. Batch normalization layers can effectively prevent network overfitting and improve training speed.

For CHMM, the parameters to be trained included the transition probability. It corresponded to two rounds of the forward and backward algorithms: once in the free-running phase (f), which corresponds to all paths without considering the labels, and once in the clamped phase (c), which is a labeled joint probability calculation. We used CML estimation to update the parameters. CML makes the probability of the free-running phase as close as possible to that of the clamped phase by adjusting the model parameters so as to achieve the desired training probability parameters. In order to facilitate calculations such as gradient descent, the negative likelihood logarithm conversion was performed aas follows:

(11) $${\hat{\Theta }}^{CML}=\underset{\Theta }{argmax}P\left(y|x,\Theta \right)=\underset{\Theta }{argmax}\frac{P\left(x,y|\Theta \right)}{P\left(x|\Theta \right)}$$

(12) $$L=-logP\left(y|x,\Theta \right)=L_{c}-L_{f}$$

(13) $$L_{c}=-logP\left(x,y|\Theta \right)$$

(14) $$L_{f}=-logP\left(x|\Theta \right)$$

In the above formula, $P\left(x|\Theta \right)$ refers to the probability of the occurrence of x under the parametric model, $P\left(x,y|\Theta \right)$ is the joint probability of x,y occurring under the parametric model, i.e., the probability of x occurring when the label sequence is consistent with the state path. We implemented a gradient descent on the transition probabilities using $\frac{\partial L}{\partial \theta_{ij}}$ in each iteration, thus, achieving an update of the transition probabilities, noting that the CHMM is different from the Baum-Welch learning method of the general HMM.

### 3.7. Co-HDNNtopss Module Training

**Figure 9:**
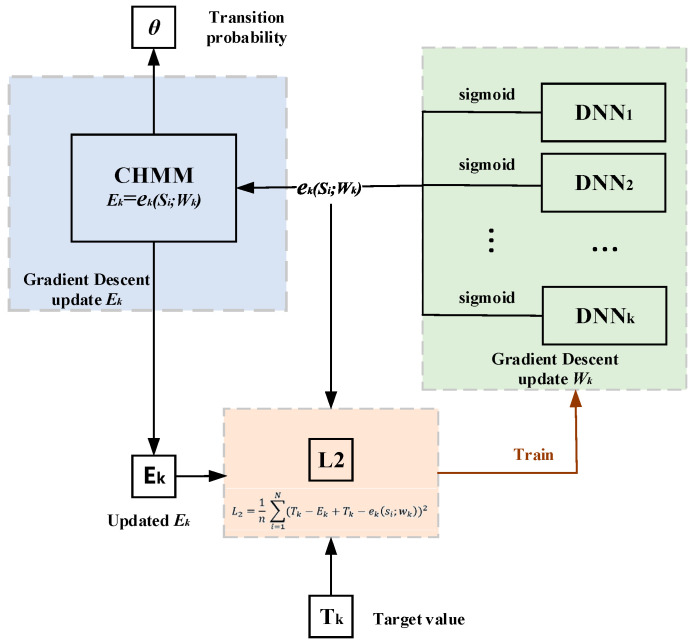
Joint training diagram. The output obtained by training the DNN model weights corresponding to each state. The output was used as initial emission probability of CHMM and the transition probability for an epoch of training. The updated emission and other parameters form a new error function used to train a new epoch of the DNNs, and this process was repeated until convergence.

Co-HDNNtopss training, the idea of joint training deep learning and CHMM, was proposed here. The flow diagram of the joint training is shown in Figure 9.

After training one epoch of weights in deep learning, we brought the output probability value $e_{k}\left(s_{i};w_{k}\right)$ of the emission network DNN_k_ into CHMM for one training iteration with the transition probability parameter in CHMM, where the output value of this emission network is gradient descent as the emission probability parameter $E_{k}=e_{k}\left(s_{i};w_{k}\right)$ in CHMM, and its gradient descent method by $\frac{\partial L}{\partial E_{k}}$ was consistent with the update of the transition probability (see Equation (15)). The updated emission probability parameter $E_{k}$ obtained after one iteration in CHMM, the target value $T_{k},$ and $e_{k}\left(s_{i};w_{k}\right)$ together form a new error calculation (see Equation (16)). Here, we regarded each state as a regression problem. The final activation function is sigmoid, and each state corresponds to a deep-learning emission network. It is worth mentioning that the outputs are the sigmoid activation regression output, with one DNN_k_ for each state. Then, we followed the commonly used MSE to establish a new loss function $L_{2}$ for each state (see Equation (17)). According to the convergence of the loss function, the gradient descent $w_{k}$ was calculated (see Equation (18)).

(15) $$E_{k}\leftarrow E_{k}-\eta \frac{\partial L}{\partial E_{k}}$$

(16) $$ERROR=T_{k}-E_{k}+T_{k}-e_{k}\left(s_{i};w_{k}\right)\;$$

(17) $$L_{2}=\frac{1}{n}\;\overset{N}{\underset{i=1}{\sum }}{\left(T_{k}-E_{k}+T_{k}-e_{k}\left(s_{i};w_{k}\right)\right)}^{2}\;$$

(18) $$w_{k}\leftarrow w_{k}-\eta \frac{\partial L_{2}}{\partial w_{k}}\;$$

This above process was repeated and then brought into CHMM training until the accuracy of the validation dataset stopped rising. At this point, the training transition probability parameters and emission probabilities were obtained. We hoped that using this approach, the two modules could achieve a joint promotion during the training so that the expectation value decreased. However, since the deep learning module was too complex and effective, a few iterations of the weights reached a reasonable value, while the transition probability parameter required many iterations to make the expected value converge, and the convergence speed and efficiency of these two were not consistent. Hence, the effect of the joint training method was not as good as the separate training, but in the subsequent improved version, we will further consider a reasonable method for training the two together to further improve the prediction accuracy.

### 3.8. Decoding

The decoder used the Viterbi algorithm, which is widely used to optimally partition the 12 states by initialization, recursion, termination, and optimal-path backtracking [56]. The optimal path for each sequence was obtained by encoding the probabilistic parameters obtained from training. Four classes of predicted structures were finally obtained, including M, H, C, and E.

## 4. Conclusions

In this study, we proposed hybrid models of CHMM and DNN to predict the topology and secondary merged structure of αTMPs from the primary sequence. The DNN mainly consists of stacked CNN and an attention-enhanced BiLSTM capturing the dependencies between contextual residuals. The statistical model CHMM extracts the correlation of labels through trained state-transition probabilities. This hybrid model not only improves the topology and secondary-structure prediction accuracy but also makes the predicted structural label strings match the actual structure according to the constraints of state-label correlation, leading to predictions that are more biologically meaningful.

Our separate training method, HDNNtopss, reflects a strong utility, with a Q4 reaching 0.779 and an MCC reaching 0.673 in the blind-test set results. Additionally, HDNNtopss achieved the best performance in the topology-prediction task compared to other state-of-the-art methods, it reached a high Q2 of 0.884 and had a strong comprehensive performance. The new method Co-HDNNtopss of joint training DNN and CHMM was also proposed, and although the accuracy was not the highest, this idea combined the two methods of training to closely promote each other. It can provide a reference for similar hybrid-model training. After discussion and analysis, the unbalanced ratio of structural label categories has been resolved.

In the future, we will choose newer datasets or reasonable sampling methods to solve the sample imbalance problem so that the joint training method can reflect a better performance capability. We will also add beta-barrel transmembrane proteins to expand the scope of our data and present the tool as a web server to make our predictor more extensive and useful.

Furthermore, we implemented HDNNtopss as a publicly available predictor for the research community. The pre-trained model and the datasets we used in this paper can be downloaded at https://github.com/NENUBioCompute/HDNNtopss.git (accessed on 10 March 2023). Finally, we sincerely hope that the predictor and the support materials we released in this study will help the researchers who need them.

## Figures and Tables

**Table 1:** Results of HDNNtopss for different window sizes on Test50.

Window Size (Residues)	Q4	TM	MCC
15	0.771	34	0.662
17	0.765	31	0.654
19	**0.779**	**38**	**0.673**
21	0.777	37	0.671
23	0.775	37	0.668

**Table 2:** Comparative benchmark of different methods in topology and secondary structure merge prediction.

Method	Class	Precision	Recall	F1-Score	Specificity	Q4	MCC	Time(S)
TMPSS [23]	M	**0.88**	0.82	0.85	0.87	0.767	0.660	89.09
	H	0.65	**0.71**	0.68	0.78			
	C	0.74	**0.77**	**0.75**	**0.84**			
	E	**0.77**	0.64	0.70	0.82			
HDNNtopss	M	0.84	0.89	**0.86**	**0.88**	**0.779**	**0.673**	**54.32**
	H	0.69	**0.71**	**0.70**	**0.79**			
	C	**0.78**	0.69	0.73	0.81			
	E	0.72	**0.73**	**0.72**	**0.86**			
Co-HDNNtopss	M	0.75	**0.93**	0.83	0.85	0.730	0.602	79.75
	H	**0.75**	0.50	0.60	0.72			
	C	0.68	0.74	0.71	0.81			
	E	0.75	0.37	0.50	0.68			

**Table 3:** Comparative benchmark of different methods in topology prediction.

Method	Q2	MCC	TM
TOPCONS [37]	0.850	0.692	32
CCTOP [38]	**0.884**	0.761	39
PolyPhobius [39]	0.855	0.708	34
OCTOPUS [19]	0.843	0.676	33
SPOCTOPUS [20]	0.834	0.656	27
SCAMPI_MSA [40]	0.836	0.663	32
DeepTMHMM [22]	0.878	0.746	**40**
Phobius [41]	0.834	0.659	27
Philius [42]	0.845	0.683	33
HDNNtopss	**0.884**	**0.763**	38
Co-HDNNtopss	0.840	0.695	31
TMPSS [23]	0.882	0.755	36

**Table 4:** Comparative benchmark of different methods for secondary-structure prediction.

Method	Q3	MCC	SOV
JPred4 [13]	0.830	0.641	74.8%
SSpro5 [43]	**0.937**	**0.863**	**88.8%**
Spider3 [44]	0.880	0.723	82.8%
PSIPRED4.0 [45]	0.866	0.696	79.3%
MUFOLD-SS [21]	0.861	0.684	79.5%
DeepCNF [46]	0.851	0.680	77.2%
Porter 5 [47]	0.883	0.734	83.72%
TMPSS [23]	0.868	0.700	81.5%
HDNNtopss	0.868	0.695	75.2%
Co-HDNNtopss	0.835	0.622	71.4%

## Data Availability

Data is contained within the article.
